# Early evidence of stone tool use in bone working activities at Qesem Cave, Israel

**DOI:** 10.1038/srep37686

**Published:** 2016-11-25

**Authors:** Andrea Zupancich, Stella Nunziante-Cesaro, Ruth Blasco, Jordi Rosell, Emanuela Cristiani, Flavia Venditti, Cristina Lemorini, Ran Barkai, Avi Gopher

**Affiliations:** 1 Department of Archaeology, Tel-Aviv University, Institute of Archaeology, POB 39040, 69978, Tel Aviv, Israel; 2 Scientific Methodologies Applied to Cultural Heritage (SMATCH), ISMN-CNR c\o Dept. of Chemistry, “Sapienza” Università di Roma, Rome, Italy; 3 Centro Nacional de Investigacìon sobre la Evolucìon Humana (CENIEH), Paseo Sierra de Atapuerca 3, 09002, Burgos, Spain; 4 Àrea de Prehistòria, Universitat Rovira i Virgili (URV), Avinguda de Catalunya, 35, 43002, Tarragona, Spain; 5 IPHES; Institut Català de Paleoecologia Humana i Evoluciò Social, Campus Sescelades URV (Edifici W3), 43007, Tarragona, Spain; 6 McDonald Institute for Archaeological Research, University of Cambridge, United Kingdom; 7 Department of Classics, Sapienza University of Rome, Rome, Italy

## Abstract

For a long while, the controversy surrounding several bone tools coming from pre-Upper Palaeolithic contexts favoured the view of *Homo sapiens* as the only species of the genus *Homo* capable of modifying animal bones into specialised tools. However, evidence such as South African Early Stone Age modified bones, European Lower Palaeolithic flaked bone tools, along with Middle and Late Pleistocene bone retouchers, led to a re-evaluation of the conception of *Homo sapiens* as the exclusive manufacturer of specialised bone tools. The evidence presented herein include use wear and bone residues identified on two flint scrapers as well as a sawing mark on a fallow deer tibia, not associated with butchering activities. Dated to more than 300 kya, the evidence here presented is among the earliest related to tool-assisted bone working intended for non-dietary purposes, and contributes to the debate over the recognition of bone working as a much older behaviour than previously thought. The results of this study come from the application of a combined methodological approach, comprising use wear analysis, residue analysis, and taphonomy. This approach allowed for the retrieval of both direct and indirect evidence of tool-assisted bone working, at the Lower Palaeolithic site of Qesem Cave (Israel).

*Homo sapiens’* supposedly exclusive manufacture of specialised tools made from modified animal bones, along with other aspects such as art and specialised hunting weapons, has led to the definition of a clear behavioural and cognitive boundary between *H. sapiens* and the other species of the genus *Homo*^1^.

However, numerous evidence, coming from South Africa and Europe, suggests that the exploitation of modified animal bones should be viewed as an expression of a much older behaviour. Some of the oldest evidence relating to the use of modified animal bones comes from several Early Stone Age South African contexts^2^,^3^,^4^,^5^,^6^. Animal bones were used by early hominids for termite foraging at the sites of Swartkrans and Sterkfontein, dated between 1.8 and 1 mya^2^,^3^. Analysis of the bone tools found at the site of Drimolen^3^,^4^, dated between 2 and 1.5 mya, provided similar results. As in the cases of Sterkfontein and Swartkrans, the use wear identified on the tips of the tools suggests their use for digging soil, most likely to be associated with termite foraging. Evidence of intentional bone modification has been recorded at Swartkrans where the tips of several horn cores bear traces of intentional shaping through grinding activities^5^. Furthermore, at Broken Hill (Kabwe) in Zambia, several bone tools were discovered which bear evidence of shaping by cutting with stone tools and subsequent polishing; these tools are attributed to the early Middle Stone Age^6^.

Other examples of tools made from modified animal bones include flaked bone tools, such as bifaces made from flaked elephant bones that have been found in several Acheulean contexts, bone flakes and bone retouchers, unearthed in numerous Middle and Late Pleistocene sites^7^,^8^,^9^,^10^,^11^,^12^,^13^,^14^,^15^. Further evidence of specialised bone tool production comes from the Middle Palaeolithic sites of Pech de l’Azé I and Abri Peyrony in France. In these sites, animal ribs were shaped by Neanderthals to create bone smoothers (*lissoirs*), which were used specifically to process hide^16^.

Here we present evidence related to the processing of animal bones, unrelated to dietary purposes and performed using specific actions and specific stone tools. This evidence originates from ongoing excavations at the Middle Pleistocene site of Qesem Cave in Israel and has not yet been observed in any other context as old as this. Our evidence includes two flint tools, which bear use wear associated with bone working and preserved bone residues on their edges, and a broken fallow deer tibia exhibiting a non-dietary sawing mark. The evidence presented here is at least 300 kya old—possibly closer to 400 kya^17^,^18^^—^and is potentially some of the earliest evidence of deliberate stone tool-assisted bone working associated with non-dietary purposes.

## Results

The Middle Pleistocene site of Qesem Cave in Israel is dated between 420 and 200 kya and is culturally associated with the late Lower Palaeolithic Acheulo-Yabrudian Cultural Complex (AYCC). Ongoing research at the cave provides a wealth of well-preserved evidence for innovative behaviours throughout the sequence, including stone tools technology and use^19^,^20^,^21^, subsistence economy^22^,^23^, and site organisation^24^,^25^, possibly practised by a new hominin lineage^26^.

## Use Wear Analysis

Ongoing functional analysis, performed by means of use wear and residue analyses (for details, see SI 1 and 2), on Quina and demi-Quina scrapers unearthed at the site, provided strong evidence of bone processing on at least one Quina scraper (QC-D/7b-1085-1090) and one demi-Quina scraper (QC-E/8b-950-955). Here we present the observations and analysis of these two flint implements.

**Figure 1:**
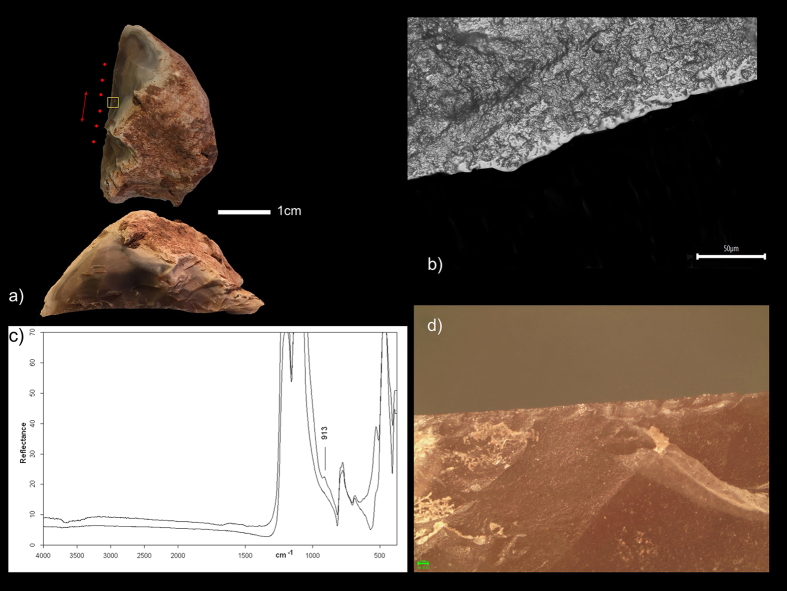
(**a**) Specimen QC-D7b-1085-1090 from Qesem Cave, the yellow square indicates the Micro-FTIR sampling point; (**b**) smooth flat polish, associated to bone working, developed over both the ventral and dorsal surface of the tool; (**c**) Micro-FTIR spectra of the tool; (**d**) edge damage located over the dorsal surface the tool associable to hard material processing.

QC-D/7b-1085-1090 (Fig. 1a) is a flint Quina scraper with an abrupt, straight edge exhibiting a scale-stepped invasive retouch. The damage observed on the tool’s edge consists of close regular step scars located both on the ventral and dorsal edge surfaces associated with an overall high degree of edge rounding (Fig. 1d). Smooth flat polish, exhibiting an oblique unidirectional orientation, is present on the tool’s edge, and on both the ventral and dorsal surfaces (Fig. 1b).

**Figure 2:**
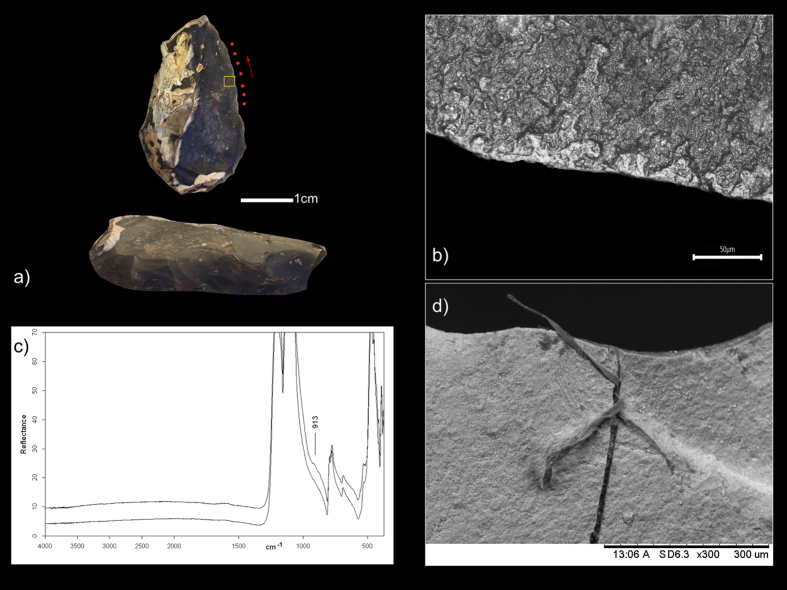
(**a**) Specimen QC-E8b-950-955 from Qesem Cave, the yellow square indicates the Micro-FTIR sampling point; (**b**) smooth flat polish, associated to bone working, developed over both the ventral and dorsal surface of the tool; (**c**) Micro-FTIR spectra of the tool; (**d**) SEM image of the collagen fibre found over the ventral surface of the tool.

QC-E/8b-950-955 (Fig. 2a) is a demi-Quina flint scraper with a thin, straight edge exhibiting a scale-stepped retouch. The damage affecting the tool’s edge consists of step scars located both on the ventral and dorsal edge surfaces, along with an overall high degree of edge rounding. Several snapped edge areas are present as well. The micro wear identified consists of a smooth flat polish located on both the tool’s ventral and dorsal surfaces (Fig. 2b). The edge damage and micro wear observed on the working edges of both tools are clearly related to bone working performed with a longitudinal motion.

## Residue Analysis

Using Micro Fourier Transform Infra-Red (Micro-FTIR) spectroscopy we were able to identify preserved bone residue on the working edges of both tools (for details, see Supplementary Information). Hydroxyapatite, representing the mineral component of bone, was found on the dorsal surface of the edges of QC-D/7b-1085-1095 (Fig. 1c) and QC-E/8b-950-955 (Fig. 2c).

In both cases the Micro-FTIR spectrum of the edge’s dorsal surface shows a shoulder on the low frequency side of the Si-O stretching mode (~913 cm^−1^); this suggests the presence of bone micro residues, as it is attributable to the PO3= stretching mode of calcium phosphate (apatite), which constitutes the bone’s mineral component.

Residues morphologically comparable to bone tissues have also been identified, via microscope, on the edge and on the surfaces of QC-E/8b-950-955.

**Figure 3:**
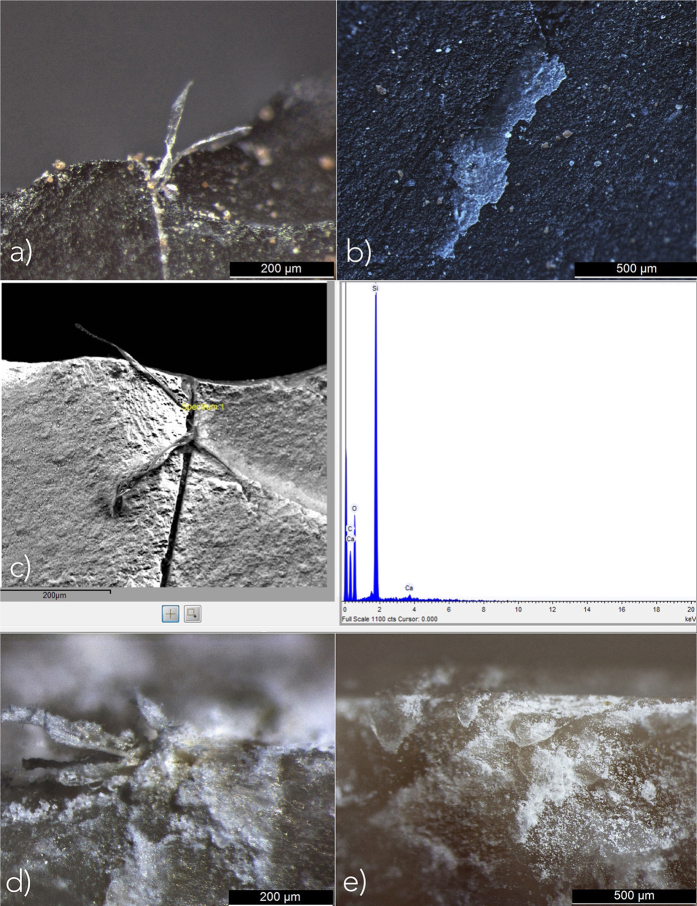
(**A**) Collagen fiber trapped in a small fracture of the edge of the archaeological stone tool; (**B**) opaque patch of residue filling micro-depressions on the surfaces of the archaeological stone tool; (**D**) and (**E**) fibers and globular residual concretions packed along the edge of experimental tools used for bone sawing.

These residues are represented by: (a) whitish elongated fibres trapped in an old fracture along the edge of the tool (Fig. 3a); (b) numerous whitish opaque patches with a corrugated and sometimes cracked appearance, filling the micro-depressions which characterise both the surfaces and the edge of the tool (Fig. 3b); and (c) white globular concretions of glossy appearance, packed into micro-fractures along the edge of the tool alone. All residues are birefringent when polarised light is used. Furthermore, it was possible to distinguish and characterise collagen fibres and bone mineral particles within the residues, using an SEM equipped with an EDS probe (Figs 2d and 3c).

Additional bone processing-related use wear and preserved bone residues have been identified on small sharp flint items produced via a form of recycling^19^,^21^,^27^, recovered from Qesem Cave following similar analytical procedures (for details, see Supplementary Information, in particular Supplementary Figure S3 and Supplementary Table S3).

## Taphonomic Analysis

**Figure 4:**
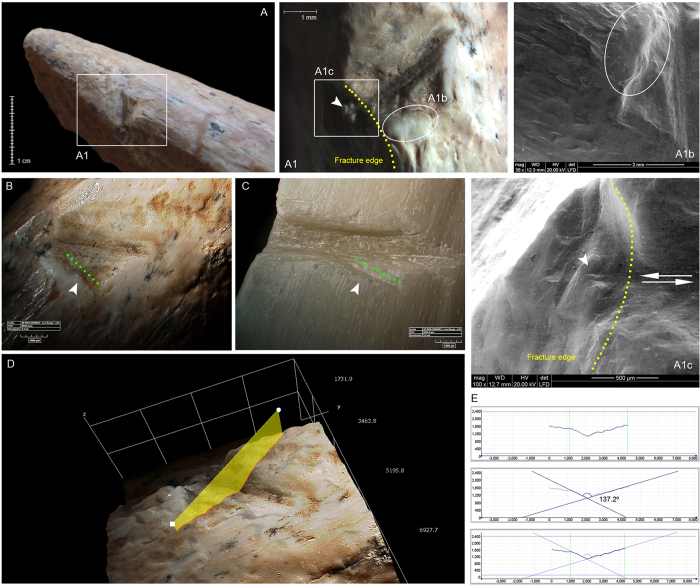
Specimen QC-F9c-735-740 from Qesem Cave showing a sawing mark (**A**) with striations on one of the fractured edges (marked by an arrow in A1 and A1c), internal micro-striations (**A1**) and certain degree of polishing and rounding (**A1b**). Note that the buffing is especially pronounced on the mark and the area around it (**A, A1, B**). The dashed green line and arrow in the picture B indicate possible secondary cuts corresponding to unintentional mistakes of accuracy similar to those observed experimentally (**C**). The image D shows a 3D reconstruction of the cortical area where the sawing mark is located (KH-8700 3D Digital Microscope), and the points used for making the section and calculating the angle (**E**).

In addition to the lithic evidence, we present a bone specimen that reinforces our hypothesis of bone working activity, performed using specific stone tools. The bone specimen from square F/9c-735–740 cm below datum is a distal tibia shaft fragment from a fallow deer. While this bone comes from a higher elevation within the sequence than the two artefacts discussed above, it is still relevant as it was associated with the same (AYCC) cultural context and with the same techno-typological flint assemblage as the previously described flint tools. The broken tibia is most probably part of a fallow deer limb that was brought to the cave, processed for food, discarded, and later modified with a flint tool. It shows a set of concentrated and overlapping short and deep incisions on the bone surface, best described as a sawing mark (Fig. 4). These incisions correspond to a repeated back-and-forth movement, during which the edge of a flint tool remained in continuous contact with the bone surface. These can be interpreted as sawing marks since they also show the characteristic V-shaped section with a slight flat bottom, oblique delineation, and internal micro-striations arranged lengthwise and parallel to the main movement axis.

The breakage characteristics (outline, fracture angle, and edge) of this bone specimen indicate a green (fresh) stage breakage. Nevertheless, jagged textures are also observable on two transverse and longitudinal breakage planes of the bone, indicating that certain post-depositional processes (including different types of pressure loading, such as trampling and/or soil compaction) may have affected it^28^. In addition, one cortical scar, documented near the longitudinal bone edge, was likely produced during bone breakage for marrow extraction.

The sawing mark is partially located on one of the bone breakage planes (Figs 4A1 and A1c), and thus had to have been generated after bone breakage. When a nutritional purpose for the processing is the case, the defleshing (and associated cut marks) takes place before bone breakage for marrow extraction. Once the bone is fragmented, little meat, if any, remains, and no further cutting is necessary. Thus, the most likely explanation in this case is that the observed deep sawing mark is the result of a non-dietary action that occurred after the bone had been de-fleshed and marrow-processed for consumption. It is worth noting that we also detected a certain degree of polishing and rounding linked to taphonomic processes which affected the whole bone, such as light sedimentary abrasion by water flow^29^. In the case of the specimen presented here, this alteration is especially evident on the sawing mark and the area around it (Figs 4A1, A1b, and Fig. 4B). We suggest that this relates to the presence of previous polishing in this area as a result of the sawing action, which was later augmented by the general post-depositional processes that acted upon it.

In order to further investigate the sawing mark identified on the archaeological specimen F/9c-735-740 a dedicated experimental framework has been applied, utilising different types of flint tools to cut through both fresh and dry bones (for details, see Supplementary Information). The results of our experimental framework indicate that the markings produced by bidirectional movements with angles that tend always to be similar, around 90°, regardless of the tool used and the condition of the bone. However, exploring the results in greater detail, some essential differences can be observed. The main difference concerns the resulting polishing and rounding of the cortical surfaces. The markings produced on dry bones are usually accompanied by a more pronounced degree of buffing, which exceeds the immediate boundaries of the mark and which can sometimes be accompanied by slight cortical notches. The other difference is the degree of inclination and opening of the walls of the mark’s section, which depends on the type and edge of the tool used. Demi-Quina scrapers usually produce wider and more inclined V-shaped sections.

Moreover, analysis was performed on the polish developed on the experimental items, taking into account the type of flint tool used and the state of the bone when the sawing was performed. Our experimental items showed a slight polishing and rounding on the sawn area, as that observed on archaeological specimen F/9c-735-740, especially when demi-Quina scrapers were used on de-fatted (dry) bones (see Supplementary Information: in particular, Supplementary Figure S3 and Supplementary Table S3).

## Discussion

Evidence related to specific activities such as carving and the cutting or scraping of bone, most likely for the production of bone tools, is scarcely known in early Palaeolithic contexts^3^,^8^,^10^. The innovative data presented here provides the first evidence of bone working, through sawing, using a specific kind of flint tools. Our evidence is considerably different in nature from that which concerns the modification of bone through use or grinding activities, suggested by several South African findings dating back to the Early Stone Age^2^,^5^. It also clearly differs from the shaping of tools (or flake knapping) of large animals’ bones, performed through flaking (e.g., using a hammerstone^30^,^31^), well known from the Lower Palaeolithic Acheulean.

The results of this study allow us to argue that at Qesem Cave, hominins were bringing selected body parts of hunted game to the cave and, after the meat, fat, and marrow were consumed, they occasionally used the discarded animal bones for non-dietary purposes. Had the bone been an isolated find, we would not easily rule out the possibility that the sawing mark was made accidentally. Yet with the current evidence of bone working use-wear and bone residues on flint tools and the sawing mark, we can state that the Qesem hominins used the bone for a non-nutritional activity and this fact can be related to the inclusion of new materials in their *chaîne opératoire*. The data presented here represents an innovative behaviour, practised between 420 and 300 kya, possibly the oldest evidence related to intentional non-dietary modification of bone through the use of specific stone tools. Moreover, our results are in perfect accordance with additional evidence related to a non-dietary exploitation of hard animal materials recorded at the site – i.e., the use of bone fragments as bone retouchers^12^.

In summary, the outstanding preservation and the application of an integrated multidisciplinary approach allowed us to identify direct and indirect evidence of bone working using stone tools at this early date. This demonstrates that the technological knowledge traditionally regarded as an expression of the Middle-Upper Palaeolithic *sapiens* cognitive sphere – i.e., the production of objects made of animal bones – was present at Qesem Cave. Our finds are significant to the debate of whether pre-*sapiens* hominins mastered bone tool production. Moreover, our results shift the debate significantly back in time, necessitating a reassessment of the evidence considered to document behavioural modernity^1^.

## Methods

The flint objects were analysed throughout the adoption of both low and high power approaches^32^. In our work, a Nikon SMZ stereomicroscope, capable of magnification up to 7.5x and equipped with fibre optic lighting, was used to analyse edge damage; a Nikon Metallurgical microscope, capable of magnification up to 500x and with reflected lighting, was used to analyse micro wear. The infrared spectra of the stone tools were collected with a Bruker Optic Alpha-R portable interferometer with an external reflectance head covering a circular area approximately 5 mm in diameter. The investigated spectral range was 7500–375 cm^−1^ at a resolution of 4 cm^−1^ and 250 scans or more. The specimens were analysed both before and after being washed, along with a sample of the sediments in which they were embedded (see Supplementary Figure S2).

Residues were observed *in situ* using a Leica 205 C stereo-microscope with LED lighting at magnifications from 10x to 165x. The nature of the residues was interpreted on the basis of their morpho-qualitative features (colour, appearance, inclusions, consistency, birefringence, etc.), through archaeological comparison^33^,^34^,^35^,^36^ as well as by evaluating a collection of comparative experimental residues. In particular, our reference collection included residues produced while using flint implements to work bone and antler, natural as well as ochre-stained hide, tendons, wood, bark, siliceous plants, and adhesive compounds (e.g. beeswax, resin, bitumen, animal glues, etc.) used for hafting. SEM-EDS analysis of the residues was performed using a Hitachi TM3000-Tabletop Scanning Electron Microscope equipped with SWIFT ED3000 EDS probe.

The fossil and experimental bones were analysed using a stereo light microscope, with a magnification of up to 120x and equipped with an oblique cold light source, and an analytical FEI QUANTA 600 Environmental Scanning Electron Microscope (ESEM), at a magnification of up to 300x operating in the low vacuum mode. The specimens were then examined with a KH-8700 3D Digital Microscope, which uses high-intensity LED optics with a full HD monitor to reconstruct three-dimensional surfaces. The criteria used to diagnose the sawing marks conformed to previously reported modifications in the taphonomic literature. Bone breakage planes were analysed in terms of outline, fracture angle, and edge, according to the criteria described by Villa & Mahieu^28^. Post-depositional alterations were also analysed and included manganese oxide precipitation, surface geochemical alteration, root etching, polishing, and rounding.

## Additional Information

**How to cite this article**: Zupancich, A. *et al*. Early evidence of stone tool use in bone working activities at Qesem Cave, Israel. *Sci. Rep.* **6**, 37686; doi: 10.1038/srep37686 (2016).

**Publisher's note:** Springer Nature remains neutral with regard to jurisdictional claims in published maps and institutional affiliations.

## Supplementary Material

Supplementary Information
