# Corrigendum: Early evidence of stone tool use in bone working activities at Qesem Cave, Israel

**DOI:** 10.1038/srep46589

**Published:** 2017-05-03

**Authors:** Andrea Zupancich, Stella Nunziante-Cesaro, Ruth Blasco, Jordi Rosell, Emanuela Cristiani, Flavia Venditti, Cristina Lemorini, Ran Barkai, Avi Gopher

Scientific Reports 6: Article number: 37686; 10.1038/srep37686 published online: 11 25 2016; updated: 05 03 2017.

In the Supplementary Information file originally published with this Article, Figure S3 was elongated and of poor quality. This error has been corrected in the Supplementary Information that now accompanies the Article.

