# Supplementary material for: Early evidence of stone tool use in bone working activities at Qesem Cave, Israel
Source: Sci Rep. 2016 Nov 25;6:37686. doi: 10.1038/srep37686 (PMC5122879; doi:10.1038/srep37686)
Supplement: Supplementary Information [file srep37686-s1.pdf]

Early evidence of stone tool use in bone working activities at Qesem Cave, Israel

Supplementary Material

Andrea Zupancich[1*], Stella Nunziante-Cesaro[2], Ruth Blasco[3,1], Jordi Rosell[4,5], Emanuela Cristiani[6], Flavia Venditti[7], Cristina Lemorini[7], Ran Barkai[1] & Avi Gopher[1]

[1]Department of Archaeology, Tel-Aviv University, Institute of Archaeology, POB 39040, 69978 Tel Aviv, Israel.
[2]Scientific Methodologies Applied to Cultural Heritage (SMATCH), ISMN-CNR c\o Dept. of Chemistry, "Sapienza" Università di Roma, Rome, Italy.
[3]Centro Nacional de Investigacìon sobre la Evolucìon Humana (CENIEH), Paseo Sierra de Atapuerca 3, 09002 Burgos, Spain.
[4]Àrea de Prehistòria, Universitat Rovira i Virgili (URV), Avinguda de Catalunya, 35, 43002 Tarragona, Spain
[5]IPHES; Institut Català de Paleoecologia Humana i Evoluciò Social, Campus Sescelades URV (Edifici W3), 43007 Tarragona, Spain.
[6]McDonald Institute for Archaeological Research, University of Cambridge, United Kingdom.
[7]Department of Classics, Sapienza University of Rome, Rome, Italy

*andreaz@post.tau.ac.il

1. *Use Wear Analysis Methodology*

The objects were analysed through the adoption of both low and high power approaches[1,2,3]. The difference between these two approaches relies on the equipment used to perform the analysis. In our work we made use of a Nikon SMZ stereomicroscope, capable of magnification up to 7.5x, and a Nikon Elite Metallurgical microscope, capable of magnification up to 500x.
After their first Micro-FTIR analysis the objects were washed by hand with fresh water and in an ultrasonic tank with deionised water.
We analysed both the edge damage and the micro wear present on the tools. Edge damages, typified by fractures, rounding, and snapping of the edge, have been categorised on the basis of their characteristics (see **Table S1**). Fractures are defined by their initial and terminal portions, along with their distribution and orientation. Edge rounding is defined by its degree.
Micro wear such as polishes, abrasions, and striations, along with the surface micro rounding, are described on the basis of their features (see **Table S1**). Polish is described and interpreted on the basis of its topography and texture, along with its orientation and distribution. Striations are defined by their morphology, orientation, location, depth, width, and bottom. The surface micro rounding is described on the basis of its degree.

2. Micro-FTIR Methodology

The infrared spectra of the stone tools were collected with a Bruker Optic Alpha-R portable interferometer with an external reflectance head covering a circular area of about 5 mm of diameter. The investigated spectral range was 7500-375 cm^{-1} at a resolution of 4 cm^{-1} and 250 scans or more. Prior to washing, the spectra of many points located at the working edge or in the inner part of each sample were collected. For comparative purposes, the same points were then analysed in the same experimental conditions following washing.
Furthermore, the sediment present beneath the tools, when sampled, was spectroscopically examined using the Diffuse Reflectance Infrared Fourier Transform (DRIFT) spectroscopy module. For the micro-FTIR analyses, very small amounts of the samples were dispersed in potassium bromide (KBr, FTIR grade purity, Fluka) in excess at different concentrations (sample\KBr:1\100 to sample\KBr:1\1000) and studied, comprising 250 scans or more in the same spectral range and resolution.
By way of example, we investigate here the spectral behaviour of the item E8b-950-955.
Figure S1 shows the DRIFT spectrum of the sediment beneath the item; Supplementary Figure S2 compares the reflectance spectra of a ventral point post-washing (a) and a dorsal point pre- and post-washing (b and c respectively). Both figures report only the spectral range in which infrared active bands were detected (4000 – 400 cm^{-1}). All absorption bands observed have been collected in a table with the proposed assignment. In the DRIFT spectrum of the sediment (Supplementary Figure S1) the intense band at 1035 cm^{-1} can be attributed to the Si-O stretching mode of silicates, which constitute the predominant component of the clay. Broad bands at 3373 and 1635 cm^{-1} are assigned to the O-H stretching and bending modes of water, or to the hydroxyl groups of the sediment, while the peak at 469 cm^{-1} is due to either O-Si-O or Si-O-Al bending modes. The low frequency weak vibrations at 528 and 422 cm^{-1} belong to metal oxides in trace amounts[4,5].
A high frequency shoulder of the Si-O stretching mode (~1098 cm^{-1}) and low intensity bands at 798 and 779 cm^{-1} indicate the presence of small residues of silica species, probably structured as quartz[6].
The medium intensity of a band at 1444 cm^{-1}, the medium weak peak at 877 cm^{-1}, and the weak band at 713 cm^{-1} suggest the existence of a significant quantity of calcite ($CaCO_3$). In addition, an overtone and two combination modes of calcite were detected at 2887, 2522, and 1797 cm^{-1} respectively[7].
In the reflectance spectrum of the ventral point of the washed item (**Figure S2a**), a very intense band is detected at 1157 cm^{-1} and assigned to the Si-O stretching mode of microcrystalline silica (flint); two medium intensity peaks at 798 and 469 cm^{-1} are due to O-Si-O or O-Si-Al bending modes[5].
Higher frequency mode (1157 and 798 cm^{-1}) bands present an up-down reversal due to the reststrahlen effect[8,9]. As all features mentioned thus far can be attributed to flint alone, the spectrum suggests the absence of working or post depositional residues.
Conversely, traces of calcite were detected on the unwashed dorsal side of the item, together with a low intensity peak around 917 cm^{-1} which we confidently attributed to residues of the mineral component of bone, $CaPO_3$ (apatite)[10]

(**Figure S2b**). The bone microresidue survived the washing process (**Figure S2c**).

3. Experimental Framework

A total of 6 experimental trials were conducted in order to establish a possible link between the stone tool and the type of marking (and its associated features). Each of these trials took several variables into account (see **Table S3**), such as the object type and the condition of the bone [fresh and dry (or defatted)]. The tools used were three demi-Quina scrapers, two lateral scrapers, and one double bulb tool, all made from flint and between 35 and 65 mm in length. The bones corresponded to mid-shaft and distal shaft fragments from the tibiae of fallow deer. All specimens were collected from animals that had died from natural causes (including accidents) in the Pyrenees (Lleida, Spain), under the supervision of rangers from the Departament d'Agricultura, Ramaderia, Pesca, Alimentació i Medi Natural de la Generalitat de Catalunya (DAAM). No animals were harmed or sacrificed by the researchers and/or rangers during this project. All the markings were generated by bidirectional movements, with none lasting more than 55 seconds. The working angle of the tool on the bone varied from 75° to 90°.

The first experimental reproduction involved a demi-Quina scraper (57x40x22 mm) and a distal fragment of fresh tibia. One of the shaped straight edges was used, following a direction oblique to the major axis of the bone for 51.26 seconds, with an inclination of between 75° and 90° on one of the longitudinal edges of the bone fracture. The result was a deep and wide sawing mark with obvious internal micro striation and a "V" section with a flat bottom (**Figure S3**A). The top edges of the "V" presented angular shapes and showed a general lack of buffing or specific rounding. Slightly parallel or zig-zag secondary cut marks were also observed, which corresponded to unintentional errors of accuracy upon initiating the main marking.

The second trial involved the use of both a demi-Quina scraper (65x46x18 mm) and a medial fragment of dry tibia diaphysis. In this case, the bidirectional movement was generated by applying one of the retouched proximal edges obliquely, for 34.99 seconds, at an angle varying between 85° and 90°. The general characteristics of the acquired marking were similar to that of the previous trial, except for the larger inclination of one of the edges comprising the "V" section, due to the greater thickness of the edge used. Associated with this, a light buffing was evident on the cortical surface of the more inclined upper edge (**Figure S3B**, **S3B1**). The degree of buffing decreased towards the bottom of the marking, becoming almost non-existent between the middle and lower area.

The third trial was performed with a demi-Quina scraper (63x23x12 mm) and a distal fragment of fresh tibia. In this case, two edges of the stone tool were used, generating two independent sawing marks (**Figure S3C, S3D**). For the first mark, the non-retouched edge of the tool was used transversely, following a bidirectional movement at an angle of 85–90° which lasted 27.22 seconds. The second sawing mark was made by using the retouched edge in a similar movement, direction and angle for 39.01 seconds. In general, both marks possess the features highlighted in the first trial, excepting **Figure S3C** which shows polishing on the straighter wall of the sawing mark (**Figure S3C1**). The ESEM

image in D1 also shows details of the micro-striations on the walls of the main mark and a clear view of its flat bottom.
The fourth trial was carried out with a small lateral scraper (34x29x10 mm) and a dry medial fragment. The working angle was 90°, in an oblique direction, and the activity lasted 35.72 seconds. The resulting mark was similar in all respects to the one from trial 2, with similar buffing in the cortical area immediately next to the mark (**Figure S3E**).
A double bulb tool (45x39x10 mm) and a fresh distal fragment were used in the fifth trial. In this case, the sawing mark was generated at an angle of 75–90° for 54.12 seconds, following a transverse direction. Again, the same general characteristics observed in trial 1 were reproduced, but a narrower "V" section with a flat bottom was generated in this case (**Figure S3F**). Associated with this, some buffing can also be seen on the micro-striations of the sawing mark (**Figure S3F1**) and in the cortical area immediately next to the marking, but at no point does it reach the level of development observed in the dry bones of trials 2 and 4. Some secondary cut marks were also observed in relation to unintentional errors of accuracy.
The sixth and last trial corresponds to a lateral scraper (52x23x5 mm) and a dry distal fragment. The duration of the bidirectional movement was 24.13 seconds, with a working angle of 75-90° and an oblique direction (**Figure S3G**). In this case, a mark with a narrow "V" section was created, with slight notches at the upper edges (**Figure S3G1**) and significant buffing across the entire cortical area surrounding the marking.
The results of these experimental trials indicate that the markings produced by bidirectional movements with angles of around 90° tend always to be similar, regardless of the tool used and the condition of the bone. However, if the results are explored in more detail, some essential differences can be observed. The main difference concerns the resulting polishing and rounding of the cortical surfaces. The markings produced on dry bones are usually accompanied by a more pronounced degree of buffing, which exceeds the immediate boundaries of the mark and which can sometimes be accompanied by slight cortical notches, as in the case of trial 6. The other difference is the degree of inclination and opening of the walls of the mark-section, which depends on the type and edge of the tool used. Demi-Quina scrapers usually produce wider and more inclined V-shaped sections than those produced by lateral and double bulb ones.

4. *Use Wear Analysis of the Recycling Tools from Qesem Cave*

The habit of recycling something that is no longer of use with the aim of producing a new object is not just a modern phenomenon but can also be found in prehistoric communities [11]
At Qesem Cave this attitude is expressed by recycling old artefacts into cores for the production of new blanks; this behaviour is recognisable in all archaeological layers.
With this lithic production, the inhabitants of the cave processed different types of materials, such as meat, wood, and hide, mainly through a longitudinal motion associable with cutting activities [12].

Contact with fresh meat often results from the treatment of animal body portions during butchery activities for dietary purposes. During this process, it is possible for the tool's edge to come into contact with bone, especially during the disarticulation stage.
From a functional point of view, this behaviour is well established by the combination of two use wear traces along the functional edge of the utilised objects: one is related to meat and the second is related to fresh bone.
The evidence of contact with fresh tissues is given by a very sheen polish with a rough texture and a greasy topography. Within the distribution of this trace, bone is easily recognisable through the evidence of a smooth and flat polish on the protruding point of the flint surface.
Since the contact with bone during the butchery activity can be sporadic or even not verifiable, bone traces can be found in association with traces of meat or be isolated with a spot-like distribution on the lithic surface. However, the combination of these two traces gives clear information about the activity carried out.
By reflecting on the characteristics and distribution of these different kinds of polishes, we can identify and distinguish the tools used to process animal carcasses from the tools used to process only bone.
Within the recycling items at Qesem Cave, at least four tools coming from the "shelf" area have been used to process fresh bone for a non-dietary purpose. The objects exhibit a well-developed smooth and flat polish along their functional edges, along with step-hinge terminating fractures and rounding.

G10c-565-570 Lateral Double Ventral Face from the Yabrudian units
This elongated tool is on the whole well preserved. It exhibits a very thin veil of white patina on its dorsal and ventral surface and a coloured-yellowish patina on its lateral section. Neither of the alterations prevented use wear and residue analysis.
The edge damages suggest that this object was used for two different motions, carried out on the same worked material. The distal edge, which exhibits a straight profile, is characterised by snap terminations along with a high rounding degree, especially on the highest portions of the surface (**Figure S4c**). The other functional edge, which follows the perpendicular axis of the flake, exhibits a medium rounding degree on the dorsal and ventral edge and step-hinge scar terminations with a wide-regular distribution and an oblique direction.
Micro wear is visible as a rough to smooth polish texture located on the protruding points of the surface where it becomes flat and matt (**Figure S4a, b**).
The polish, although well recognisable, is not extremely developed. This can be determined by the texture and the topography of the polish itself, which appears as rough and domed where it is less developed, while it becomes smooth and flat on the protruding and more developed points. The use wear present on the tool suggests its use for the processing of bone. Two motions have been performed with the tool, according to the orientation of both micro wear and edge damage: one transversal with the distal portion of the item, and the other longitudinal along the edge. The presence of bone micro residue at the frequency of 912 cm-1 has been identified on the distal edge of the ventral face through Micro-FTIR analysis (**Figure S5b**).

Furthermore, the SEM-EDS analysis made on the same ventral face and, more precisely, in the middle of the edge ventral surface, recorded a high percentage of two chemical elements that constitute bone: Phosphorus (P) and Calcium (Ca) which, combined, indicate Calcium Phosphate (**Figure S5a**). The highest percentage recorded on the organic residue (spot measure) was 10% for Phosphorus and 20% for Calcium.

F11a-645-650 Lateral Double Ventral Face from the Amudian units.
The tool was in a very high state of preservation. Any types of patina, alteration, or fractures were visible.
Both the ventral and dorsal surfaces exhibit edge damage, while the degree of rounding is low. Step and hinge scars are visible with a wide regular distribution. The direction of the step fractures is oblique (**Figure S6a**). It is not possible to define if the motion was unidirectional or bidirectional.
In this case too, the polish identified is not highly developed.
On the protruding point alone the polish appears smooth and flat, especially along the outer edge portion (**Figure S6b**). This suggests that the action was probably brief or carried out with little energy. The use wear identified suggests the exploitation of this tool in a longitudinal motion, probably short, performed on bone.
Micro-FTIR analysis highlighted a microresidue of Hydroxyapatite, a bone mineral component (PO3) represented, in the spectrum, by a shoulder at the frequency of 912 cm-1, located on the distal portion of the edge (**Figure S6c**). The other three measurements did not provide evidence of preserved microresidue.

H11a-540 Double Bulb Double Ventral Face Kombewa from the Amudian units.
This tool was well preserved, notwithstanding a light layer of white patina that affected its outer edge. A fracture, probably occurred during the tool use, is present in the middle of the functional edge, on its dorsal surface.
As a result of this fracture, use-related edge removals are visible only on a small portion of the edge. These are represented by large hinge scars with close regular distribution and a medium degree of rounding (**Figure S7a**). Edge damage is located on the edge's dorsal surface and is most likely related to contact with hard material through a transversal motion.
Edge damages are related to a well-developed smooth and flat polish, running from the outer portion of the edge to its inner surface. Due to its extent, the micro edge surface appears very rounded (**Figure S7b**).
According to our interpretation of the use wear identified, the straight and thick edge was used for scraping a bone surface, probably for a long time and with a high degree of energy. The processed bone could be extremely clean, with no trace of meat or fat, as any polish other than the bone like one was identified. The use of this object has certainly been facilitated by its size (unusual for this category of objects) and by its ergonomic shape.
The Micro-FTIR measurement performed along the preserved functional edge's dorsal surface pointed out the presence of micro residue of a bone mineral component (Hydroxyapatite) at the frequency of 912 cm-1, which accords with the bone working-related use wear previously identified in this area of the object (**Figure S7c**).

G10a-615-620 Double Bulb Double Ventral Face from the Yabrudian units.

The tool is well preserved and did not exhibit any type of post depositional alteration or patina.

On the ventral face, the functional edge is characterised by a series of snaps, within which are located some edge removals exhibiting a feather termination and a close regular distribution.

On its dorsal face, a sequence of snap and half-moon scars are present, characterised by a close regular distribution (**Figure S8a**). A medium degree of edge rounding affected the edge.

A continuous polish, exhibiting a rough to smooth texture and a flat topography, is located on both ventral and dorsal edge surfaces, in particular on its protruding points (**Figure S8b**). In no Micro-FTIR analysis was any Hydroxyapatite detected. However, two measurements taken over the edge dorsal surface indicate the presence of Adipocere. This latter is indicated by two absorptions, roughly at the frequencies of 1537 and 1573 cm-1 (**Figure S8c**).The Adipocere is an insoluble greyish-white substance produced by the decomposition of saturated fatty acids due to particular environmental conditions. A humid, hot, and anaerobic environment is perfect for the formation and preservation of Adipocere, which is able to survive for thousands of years [13,14,15].

The presence of Adipocere on the tool can be explained by three possible scenarios:

1) the worked bone could be unclean at the moment of its processing, and still covered by remains of meat and fat;
2) contact with marrow may have left traces of fatty acids over the edge;
3) the tool could have been previously used to process a material different from bone.

However, the absence of typical meat-associated use wear and the intensity of the bone polish allow us to discard the hypothesis of the use of this object for butchering, leaning instead towards a major contact with bone through mixed activities.

References

1. Keeley, L. H. *Experimental Determination of Stone Tool Uses*. (The University of Chicago Press, 1980).

2. Tringham, R., Cooper, G., Odell, G. H., Voytek, B. & Whitman, A. Experimentation in the Formation of Edge Damage: A New Approach to Lithic Analysis. *J. F. Archaeol.* 171–196 (1974).

3. Gijn, A. van. *Flint in Focus; Lithic Biographies in the Neolithic and Bronze Age*. (Sidestone Press, 2010).

4. Madejová, J. FTIR techniques in clay mineral studies. *Vib. Spectrosc.* **31,** 1–10 (2003).

5. Vaculíková, L. & Plevová, E. Identification of clay minerals and micas in sedimentary rocks. *Acta Geodyn. Geomater.* **2,** 167–175 (2005).

6. De Benedetto, E. G., Catalano, F., Sabbatini, L. & Zambonin, G. P. Analytical characterisation of pigments on pre-Roman pottery by means of spectroscopic techniques Part I: white coloured shards. *Fresenius. J. Anal. Chem.* **362,** 170–175 (1998)

7. Gunasekaran, S., Anbalagan, G. & Pandi, S. Raman and infrared spectra of carbonates of calcite structure. *J. Raman Spectrosc.* **37,** 892–899 (2006).

8. Hunt, G. R. & Logan, L. M. Variation of Single Particle Mid-Infrared Emission Spectrum with Particle Size. *Appl. Opt.* **11,** 142–147 (1972).

9. Salisbury, J. W. & Wald, A. The role of volume scattering in reducing spectral contrast of reststrahlen bands in spectra of powdered minerals. *Icarus* **96,** 121–128 (1992).

10. Acerbo, A. S., Carr, G. L., Judex, S. & Miller, L. M. Imaging the Material Properties of Bone Specimens Using Reflection-Based Infrared Microspectroscopy. *Anal. Chem.* **84,** 3607–3613 (2012).

11. Barkai, R., Lemorini, C. & Vaquero, M. The origins of recycling: A Paleolithic perspective. *Quat. Int.* **361,** 1–3 (2015).

12. Lemorini, C. *et al.* The function of recycled lithic items at late Lower Paleolithic Qesem Cave, Israel: An overview of the use-wear data. *Quat. Int.* **361,** 103–112 (2015).

13. Langejans, G. H. J. Remains of the day-preservation of organic micro-residues on stone tools. *J. Archaeol. Sci.* **37,** 971–985 (2010).

14. Nunziante Cesaro, S. & Lemorini, C. The function of prehistoric lithic tools: a combined study of use-wear analysis and FTIR microspectroscopy. *Spectrochim. Acta. A. Mol. Biomol. Spectrosc.* **86,** 299–304 (2012).

15. Solodenko, N. *et al.* Fat Residue and Use-Wear Found on Acheulian Biface and Scraper Associated with Butchered Elephant Remains at the Site of Revadim, Israel. *PLoS One* **10,** 1–17 (2015).

Figures

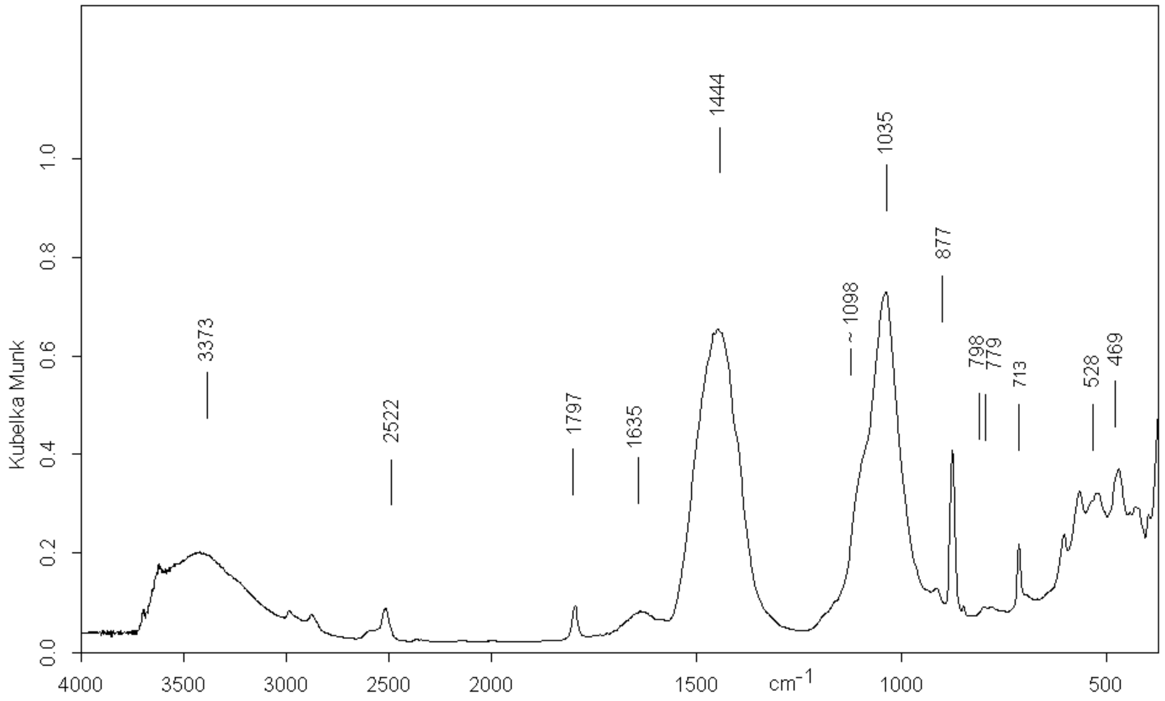

Figure S1. DRIFT spectrum of sediment of item QC/E8b-950-955.

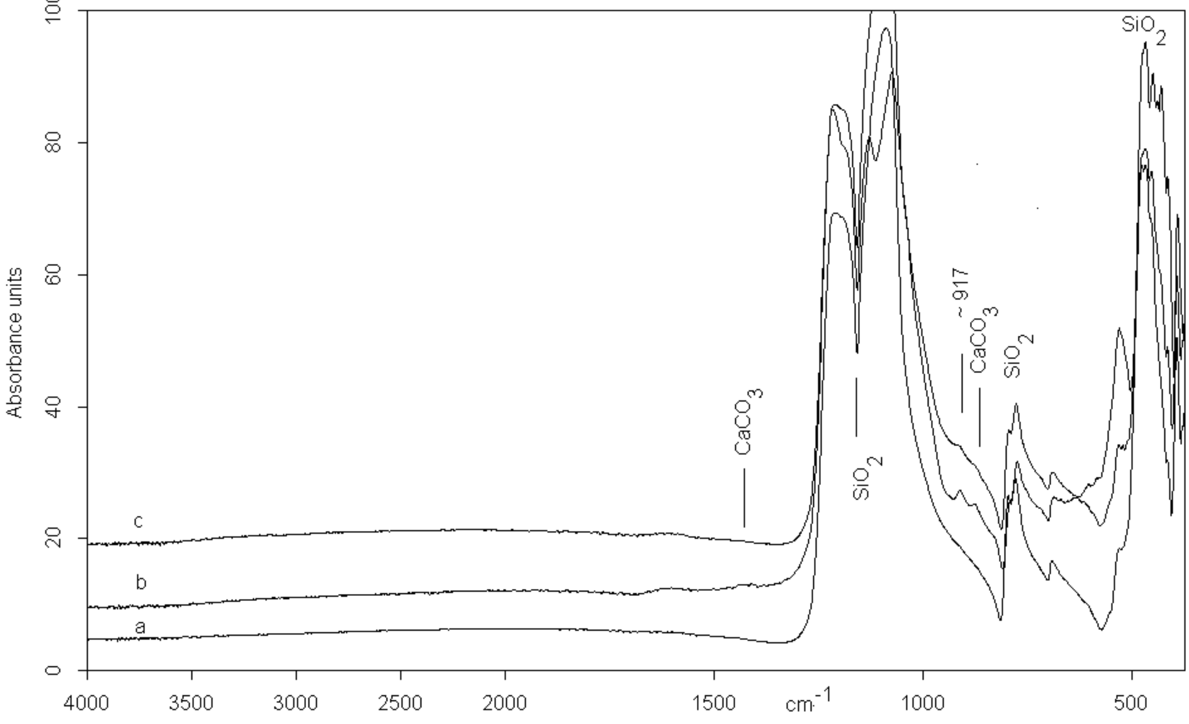

Figure S2. Reflectance spectrum of item QC/E8b-950-955: a) ventral surface sample point after washing; b) dorsal surface sample point prior to washing; c) dorsal surface sample point after washing.

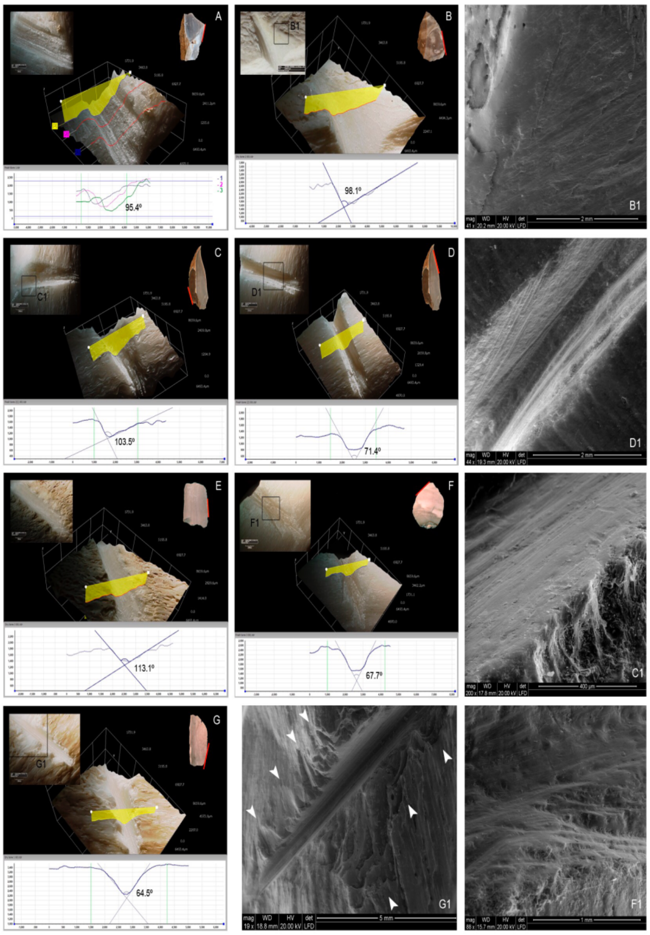

Figure S3. Experimental reproductions of sawing marks on several distal shaft fragments of tibia of fallow deer with different states of freshness: a) Mark generated by a demi-Quina scraper (57x40x22 mm) on a fresh tibia fragment; b) Mark produced by a demi-Quina scraper (65x46x18 mm) on a dry tibia shaft. Note the degree of polishing and rounding on the more inclined upper wall of the mark in the B1 ESEM image; c, d) Marks generated by a demi-Quina scraper (63x23x12 mm) on a distal fragment of fresh tibia. Note the polishing on the straighter wall of the sawing mark in the C1 ESEM image, the micro-striations on the walls composing the main mark, and the flat bottom in the D1 ESEM image; e) Mark produced by a small lateral scraper (34x29x10 mm) on a dry medial fragment; f) Mark generated by a double bulb tool (45x39x10 mm) on a fresh distal fragment. Note the polishing showing the micro-striations composing the sawing mark in F1 ESEM image; g) Mark produced by a lateral scraper (52x23x5 mm) on a dry distal fragment. In this case, slight notches were generated on the cortical surface of both upper edges of the sawing mark (G1). Red lines on lithic tools mark the cutting edge used during the experimental reproductions of sawing marks.

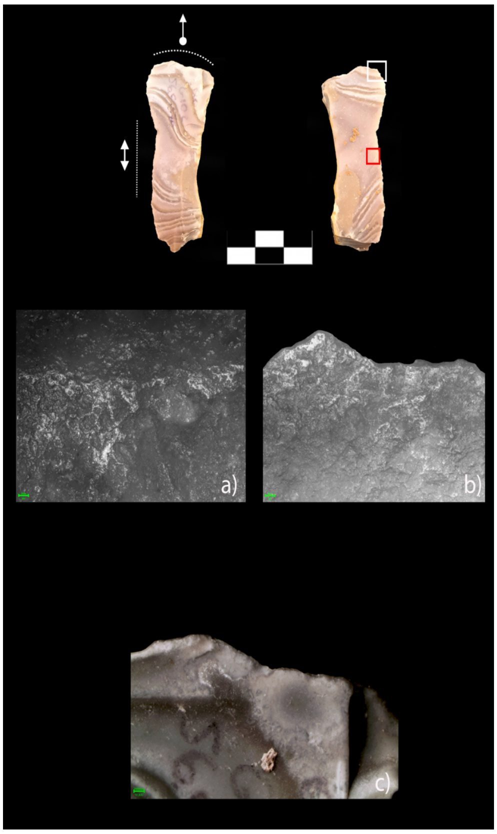

Figure S4. Item G10c-565-570; use-wear related to scraping bone: a-c) polish and edge damage located on the ventral surface face and associated with scraping bone; b) polish on the dorsal surface associated with cutting bone. Red and white square indicates, respectively, the EDS and Micro FTIR sampling points.

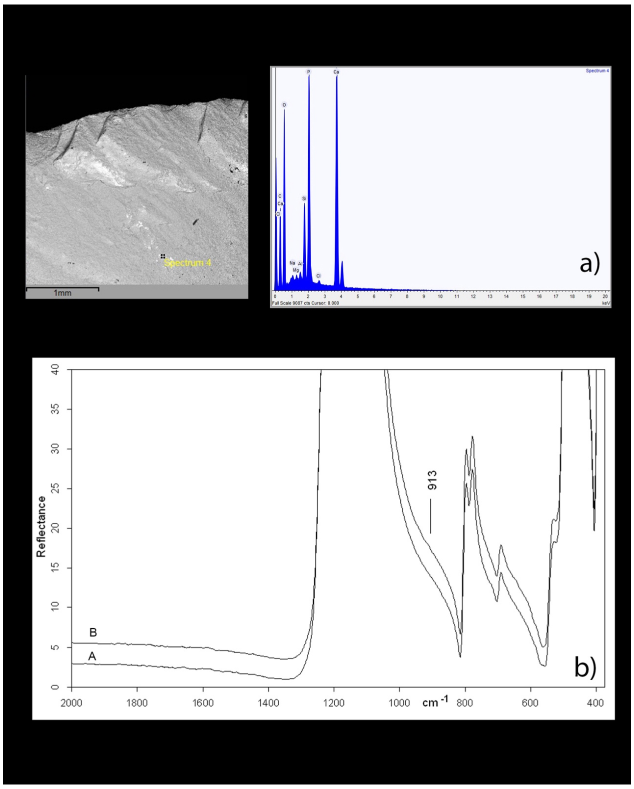

Figure S5. EDS-histogram and Micro FTIR spectrum of item G10c-565-570: a) histogram showing the high percentage of Phosphorus (P) and Calcium (Ca); b) spectrum showing the fundamental mode of pure silica (A) and the point on the ventral edge with bone microresidues (B).

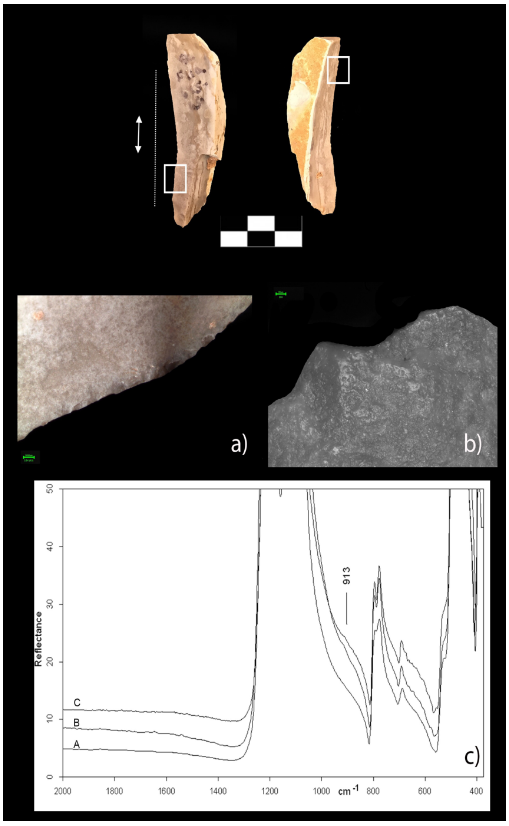

Figure S6. Item F11a-645-650; use-wear and Micro FTIR spectrum related to cutting bone: a) edge damage; b) polish on the edge ventral surface associated with cutting bone; c) Micro FTIR spectrum showing the fundamental mode of pure silica (A) and the points on the ventral and dorsal edge surfaces with bone microresidues (B). White squares indicate the Micro FTIR sampling points.

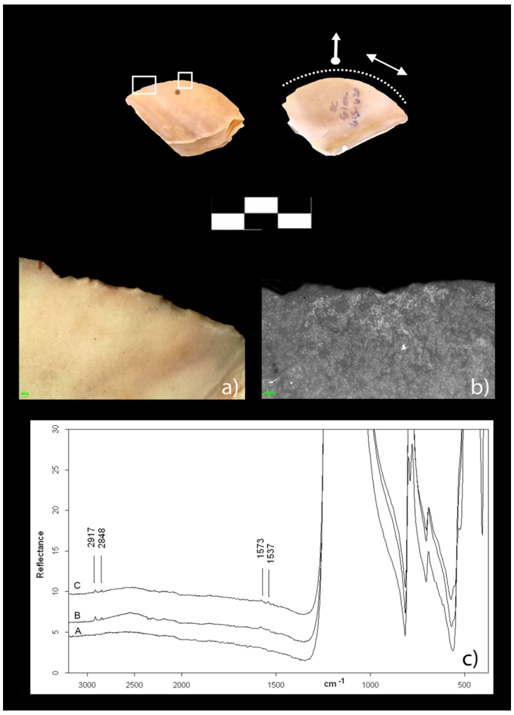

Figure S7. Item H11a-540; use-wear and Micro FTIR spectrum related to scraping bone: a) edge damage; b) polish on the ventral edge surface related to scraping bone; c) Micro FTIR spectrum showing the fundamental mode of pure silica (A) and the points on the ventral and dorsal edge surfaces with Calcium carbonate (B) and bone microresidue (C). White squares indicate the Micro FTIR sampling points

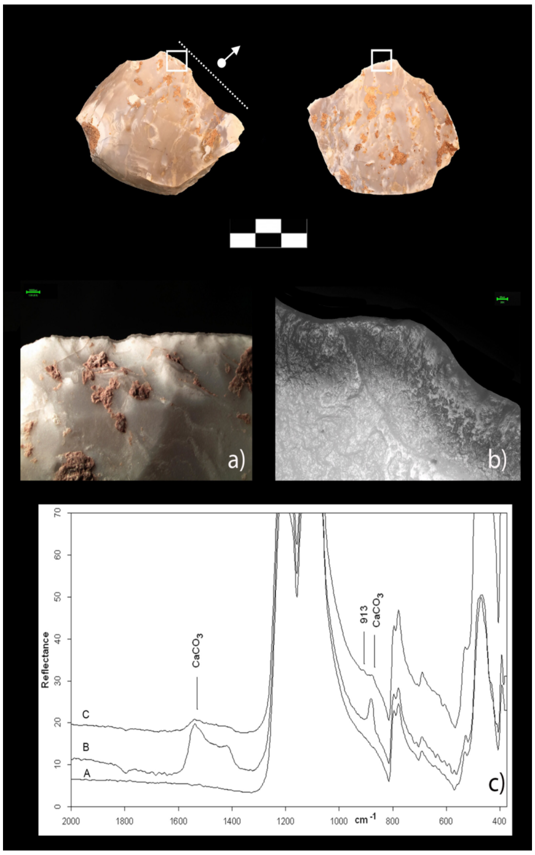

Figure S8. Item G10a-615-620; use-wear and Micro FTIR spectrum related to mixed activities: a) edge damage; b) polish located on the ventral edge surface related to mixed activities; c) Micro FTIR spectrum showing the fundamental mode of pure silica (A) and the points on the dorsal edge surface with presence of CH (organic bonds) and adipocere microresidue (C).

**Edge Damage**	**Initiation**	*Cone*	*Bending*					
	**Termination**	*Feather*	*Step*	*Hinge*				
	**Distribution**	*Close Regular*	*Close Irregular*	*Wide Regular*	*Wide Irregular*	*Overlapping*		
	**Orientation**	*Transversal*	*Longitudinal*	*Oblique Unidirectional*	*Oblique Bidirectional*			
	**Edge Rounding Degree**	*Low*	*Medium*	*High*				
**Micro Wear**	**Polish Texture**	*Smooth*	*Rough*	*Tending Smooth*				
	**Polish Topography**	*Flat*	*Domed*	*Granular*	*Melted Snow*	*Comet Shape*	*Pitted*	*Cratered*
	**Polish Distribution**	*Continuous*	*Discontinuous*	*Spot like*	*Reticulated*			
	**Polish Orientation**	*Transversal*	*Longitudinal*	*Oblique Unidirectional*	*Oblique Bidirectional*			
	**Polish Extension**	*Outer edge*	*Edge*	*Surface*	*Surface and Edge*			
	**Striation Morphology**	*Short*	*Long*	*Comet tails*				
	**Striation Orientation**	*Transversal*	*Longitudinal*	*Chaotic*	*Mixed*			
	**Striation Location**	*Ventral*	*Dorsal*	*Ventral more*	*Dorsal More*	*Ventral and Dorsal*	*Indeterminable*	
	**Striation Depth**	*Shallow*	*Deep*	*Shallow and Deep*				
	**Striation Width**	*Large*	*Narrow*	*Large and Narrow*				
	**Striation Bottom**	*Polished*	*Matt*	*Pointed*	*Corrugated*	*Grooved*		
	**Micro Rounding Degree**	*Low*	*Medium*	*High*				

Table S1. Micro Wear and Edge Damage nomenclature used to describe the identified use wear

DRIFT spectra absorption bands (cm^{-1})	Micro-FTIR spectra absorption bands (cm^{-1})	Proposed assignment
3373 ms, br		n_3 - O-H stretching of H_2O
2990 vvw		
	2916 vw	C-H stretching organic material
2887 vvw		2 n_3 $CaCO_3$
	2848 vw	C-H stretching organic material
2522 w	2520 w	2 n_2 + n_4 $CaCO_3$
1797 ms	1797 m	n_1 + n_4 $CaCO_3$
1635 m		n_2- H-O-H bend of H_2O
	1575 vvw	adipocere
	1538 vvw	adipocere
1444 s		n_3 - asymmetric CO_3 stretching of $CaCO_3$
	1157 vs	Si-O st of flint
~ 1090 sh		Si-O stretching of SiO_2 (quartz)
1035 vs		Si-O stretching of silicates
918 m	~ 917 sh	n_3 - PO_4 stretching mode (hydroxyapatite)
876 ms		n_2 - asymmetric CO_3 deformation of $CaCO_3$
798 w		O- Si-O bend of SiO_2 (quartz)
	789 m	O-Si-O bend of flint
779 w		O- Si-O bend of SiO_2 (quartz)
715 mw		n_4 -- symmetric CO3 deformation of $CaCO_3$
528 m	532 m	
469 ms	469 ms	Si-O-Si bending of SiO_2 (flint and silicates)
422 m	420 m	

Table S2. Vibrational wavenumbers and proposed assignment of absorption bands observed in DRIFT and Micro-FTIR spectra.

Tool Id	Tool					Experiment Variables					
	Object type	*Raw material*	*Measures (LxWxT) -mm*	*Edge morphology*	*Cross section*	*Duration*	*Activity*	*Bone state*	*Used edge*	*Motion*	*Angle of work*
1	demi-Quina scraper	Flint	57x40x22	Straight	Straight-Straight	51''26cs	Sawing	Fresh	Shaped - Straight	Oblique, Bidirectional	75-90º
2	demi-Quina scraper	Flint	65x46x18	Straight	Convex-Straight	34''99cs	Sawing	Dry	Shaped - Convex	Oblique, Bidirectional	85-90º
3	demi-Quina scraper	Flint	63x23x12	Convex	Convex-Straight	27''22cs (a), 39''01cs (b)	Sawing	Fresh	Not shaped - straight (a), Shaped - Convex (b)	Transverse, Bidirectional (a), Transverse (oblique), Bidirectional (b)	85-90º (a), 85-90º (b)
1	Lateral	Flint	34x29x10	Straight	Convex-Straight	35''72cs	Sawing	Dry	Not shaped - Straight	Oblique, Bidirectional	90º
2	Double bulb	Flint	45x39x10	Straight	Concave-Convex	54''12cs	Sawing	Fresh	Not shaped - Concave	Transverse, Bidirectional	75-90º
3	Lateral	Flint	52x23x5	Straight	Convex-Straight	24''13cs	Sawing	Dry	Not shaped - Straight	Oblique, Bidirectional	75-90º

Table S3. Detailed variables of the experimental framework set up in order to investigate the types of sawing mark produced by Quina, demi-Quina scrapers, and recycled items.
